# Resectable pancreatic ductal adenocarcinoma: association between preoperative CT texture features and metastatic nodal involvement

**DOI:** 10.1186/s40644-020-0296-3

**Published:** 2020-02-10

**Authors:** Wei Huan Fang, Xu Dong Li, Hui Zhu, Fei Miao, Xiao Hua Qian, Zi Lai Pan, Xiao Zhu Lin

**Affiliations:** 1 Department of Nuclear Medicine, Shanghai Jiao Tong University Medical School Affiliated Ruijin Hospital, 197 Ruijin Er Road, Shanghai, 200025 NO China; 2 Department of Radiology, Shanghai Jiao Tong University Medical School Affiliated North Ruijin Hospital, Shanghai, China; 3 Department of Radiology, Fudan University Shanghai Cancer Center, Shanghai, China; 4 Department of Radiology, Shanghai Jiao Tong University Medical School Affiliated Ruijin Hospital, Shanghai, China; 5 Institute of Medical Imaging Technology, School of Biomedical Engineering, Shanghai Jiao Tong University, Shanghai, China

**Keywords:** Texture analysis, Computer-assisted image processing, Computed tomography, Pancreatic ductal adenocarcinoma, Metastases

## Abstract

**Background:**

To explore the relationship between the lymph node status and preoperative computed tomography images texture features in pancreatic cancer.

**Methods:**

A total of 155 operable pancreatic cancer patients (104 men, 51 women; mean age 63.8 ± 9.6 years), who had undergone contrast-enhanced computed tomography in the arterial and portal venous phases, were enrolled in this retrospective study. There were 73 patients with lymph node metastases and 82 patients without nodal involvement. Four different data sets, with thin (1.25 mm) and thick (5 mm) slices (at arterial phase and portal venous phase) were analysed. Texture analysis was performed by using MaZda software. A combination of feature selection algorithms was used to determine 30 texture features with the optimal discriminative performance for differentiation between lymph node positive and negative groups. The prediction performance of the selected feature was evaluated by receiver operating characteristic (ROC) curve analysis.

**Results:**

There were 10 texture features with significant differences between two groups and significance in ROC analysis were identified. They were WavEnLH_s-2(wavelet energy with rows and columns are filtered with low pass and high pass frequency bands with scale factors 2) from wavelet-based features, 135dr_LngREmph (long run emphasis in 135 direction) and 135dr_Fraction (fraction of image in runs in 135 direction) from run length matrix-based features, and seven variables of sum average from coocurrence matrix-based features (SumAverg). The ideal cutoff value for predicting lymph node metastases was 270 for WavEnLH_s-2 (positive likelihood ratio 2.08). In addition, 135dr_LngREmph and 135dr_Fraction were correlated with the ratio of metastatic to examined lymph nodes.

**Conclusions:**

Preoperative computed tomography high order texture features provide a useful imaging signature for the prediction of nodal involvement in pancreatic cancer.

## Background

Pancreatic cancer is the fourth most common cause of death by cancer worldwide [1]. Lymph node (LN) involvement is known to be the main prognostic factor. Lymph node metastases and the ratio of metastatic to examined lymph nodes (LNR) were associated with worse disease free-survival (DFS) and overall survival (OS) in patients with resected pancreatic ductal adenocarcinoma (PDAC) [2]. Previous study showed that adjuvant chemoradiotherapy in pancreatic cancer is associated with a significant improvement of survival only in patients with LN-positive disease, while the effects of chemoradiotherapy on patients with LN-negative disease may be limited [3]. Initial imaging of PDAC is of crucial importance in the decision-making process, and accurate preoperative staging is essential to determine the best treatment modality for each patient. But for the conventional preoperative imaging (computed tomography [CT], endoscopic ultrasound [EUS], magnetic resonance imaging [MRI]), the sensitivity, specificity, positive predictive value, and negative predictive value were low for lymph node invasion [4]. In resectable PDAC, CT is not accurate overall for the prediction of nodal involvement [5]. So preoperative diagnosis of lymph node metastases is still a challenge currently.

Dual-energy CT has been widely used recently [6]. Quantitative parameters derived from dual-energy CT data have proven to be useful for the diagnosis and nodal staging in several types of tumors [7–10]. However, the value of quantitative parameters from dual-energy CT in the preoperative diagnosis of LN metastasis in patients with pancreatic cancer remains elusive. With the development of computer technology, a novel graphics analysis technology, namely, texture analysis, has been applied in medical imaging. Texture analysis can extract much more data from medical images than the naked eye by means of quantitatively analyzing grey scale distribution features, inter-pixel relations, and spatial features of images [11]. Texture features might detect distinct quantifiable phenotypic differences of tissues which cannot be assessed through a qualitative, visual evaluation of radiological images alone. Based on these texture features, it is possible to distinguish between healthy and pathologically altered tissues and also between different types of tissue. In colorectal cancer, the radiomics features from CT image were significantly associated with LN status [12]. There are few studies have focused on the nodal involvement of PDAC using texture analysis with CT image. One study [13] showed that mean attenuation value of the whole tumor (WHOLE-AV) and mean of the positive pixels variables from fine (spatial scaling factor, SSF2) to coarse (SSF6) texture levels were significantly associated with the presence of lymph nodes invasion. We hypothesized that texture features derived from contrast-enhanced images of preoperative dual-energy CT may be associated with the involvement of the LN in patients with pancreatic cancer. In this study, the quantitative parameters of primary tumor were obtained from preoperative CT images. The purpose of this study is to explore the relationship between the lymph node status and preoperative texture features on preoperative contrast-enhanced CT images of the primary tumor in PDAC.

## Methods

### Patients

**Fig. 1:**
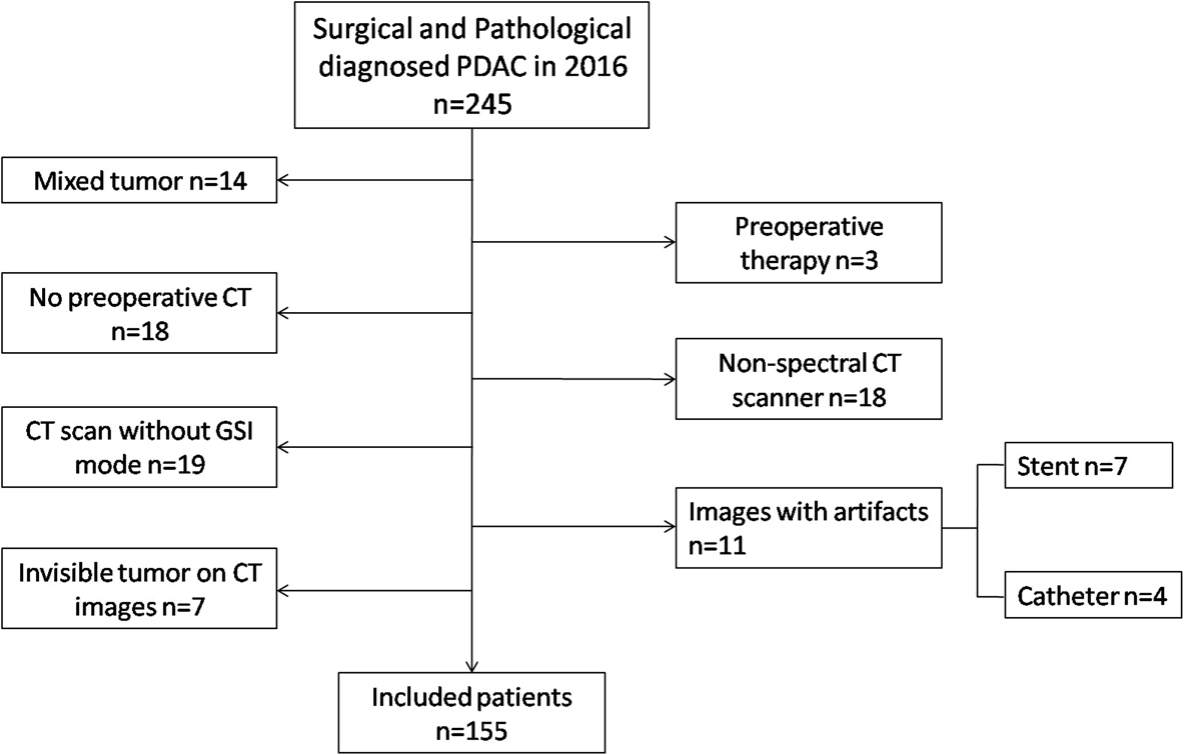
Illustration of flow diagram of included patients

The institutional review board approved this retrospective study and waived the written informed consent requirement. According to the database of pathology in 2016, a total of 245 consecutive patients with PDAC who were operated at our institution were enrolled. Patients underwent potentially curative resection (R0 and R1) with standard regional lymph node dissection. All PDAC were comfirmed by pathlogical analysis. The tumors were staged according to tumor-node-metastasis (TNM) cancer staging system (the 7th edition) by the American Joint Committee on Cancer (AJCC) and the International Union for Cancer Control (IUCC) [14]. The inclusion criteria were as follows: a. both primary tumor and regional lymph nodes surgical resections were performed and PDAC was confirmed by pathology; and b. contrast-enhanced CT images obtained on CT750 HD scanner (GE Healthcare, Milwaukee Wis, USA) using spectral imaging (GSI) mode; c. no neoadjuvant therapy before surgery. Finally, a total of 155 patients (104 men, 51 women; mean age 63.8 ± 9.6 years; range 37–83 years) were enrolled in this retrospective study (Fig. 1). Detailed pathological reports, including all resected specimens, were obtained through hospital information system (HIS). The total number of examinated lymph nodes and the number and location of the positive lymph nodes were recorded. Study subjects were divided into two groups according to lymph node status. Seventy-two cases with lymph node metastases (N+ group) and 83 cases without lymph node metastases (N- group) were used for texture analysis. According to the detail report of pathology, LNR of each case was also calculated in this study by the radiologist (XZL). LNR was calculated as the ratio of (positive number)/(total number). Among the 72 cases with positive lymph nodes, in 43 cases metastases were located in the peri-pancreatic region, in 16 cases peri-tumoral, and in 10 cases in the hepatoduodenal ligament (no.12), in 12 cases in no.14, in 6 cases in no.9, in 4 cases in no.16, in two cases in no.7 and no.5, and in one case in no.10 and no.6.

### CT imaging technique

CT examinations were performed with a 64 row detector scanner (CT750 HD) using spectral imaging (GSI) mode with fast tube voltage switching between 80kVp and 140 kVp on adjacent views during a single rotation on the late arterial phase (AP) and portal venous phase (PVP). Contrast enhanced images were obtained after administration of nonionic contrast medium (Iopamidol Injection, Iopamiro 300; Shanghai BRACCO Sine Pharmaceutical Corp. Ltd., China. Ioversol, Optiray 320; Tyco Healthcare, Montreal, Quebec, Canada) at a dose of 450 mg iodine per kilogram of body weight. All the contrast medium was injected in 30 s, followed by 20 ml saline at the same injection rate. AP images were acquired at 35 s and PVP images were acquired at 65 s after the start of contrast material administration. CT images without contrast were obtained with a tube voltage of 120 kVp, an automatic tube current of 100–500 mA, a noise index (NI) of 10, and a rotation time of 0.6 s. CT images in AP and PVP were obtained with GSI mode (tube voltage switching instantaneously between 80 kVp and 140 kVp, and a dedicated GSI protocol with similar CT dose index (CTDI) of pre-contrast scan was selected. The other CT image acquisition parameters included: (1) pitch, 1.375; (2) detector collimation of 64*0.625 mm; (3) image matrix of 512× 512; (4) FOV (field of view) of 35~40 cm; (5) Virtual monochromatic (VMC) images were reconstructed with standard algorithm and slice thickness of 5 mm (at 65 k electron voltage, keV) and 1.25 mm (at 60 keV). An adaptive statistical iterative reconstruction (ASiR, 30% for 5 mm and 40% for 1.25 mm) algorithm was employed to reduce the image noise. The aforementioned protocol was the routine protocol for preoperative staging of pancreatic cancer in our hospital. Theoretically and according to the literature, virtual monochromatic image with lower keV level had higher contrast-to-noise-ratio (CNR) and noise [15, 16]. While only limited application of noise reduction software of ASiR in spectral imaging on CT750 HD scanner (GE Healthcare, Milwaukee Wis, USA) was approved by China Food and Drug Administration in 2016, the noise reduction technique could not be applied on the virtual monochromatic images lower than 60 keV in our CT scanner then. Considering the balance of CNR and noise, 60 keV and 65 keV were adopted for pancreas then in our center. 60 keV was also used in other studies [17, 18]. So there were totally 4 image subsets (5 mm-AP-65 keV, 5 mm-PVP-65 keV, 1.25 mm-AP-60 keV, 1.25 mm-PVP-60 keV) in this study, and each image subset used the same window level and window wide.

### Feature extraction and selection by software

**Fig. 2:**
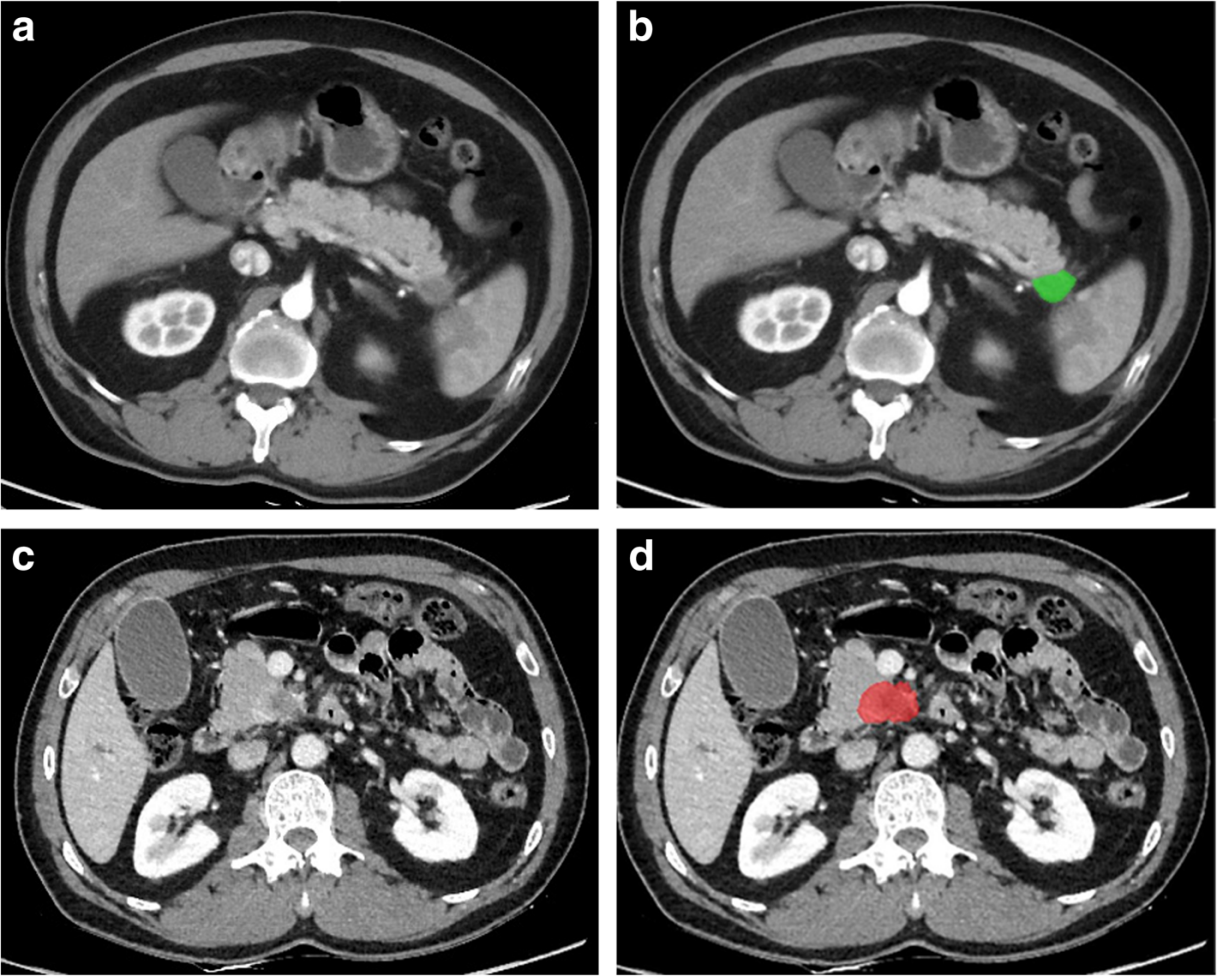
Illustration of drawing the region of interest (ROI) by MaZda software 77 years old male, pancreatic ductal adenocarcinoma (PDAC) in the pancreatic tail (green ROI), without lymph node (LN) metastases, axial virtual monochromatic (VMC) CT images at 65 k electron voltage (keV) on late arterial phase with 5 mm slice thickness (**a**, **b**). 53 years old male, PDAC in the pancreatic head (red ROI), with 1/25 metastatic LN, axial VMC CT images at 60 keV on portal venous phase with 1.25 mm slice thickness (**c**, **d**)

We conducted a texture analysis on the axial contrast enhanced VMC CT images on AP and PVP. Texture analysis was performed with MaZda statistical texture analysis software (version 4.6, available at http://www.eletel.p.lodz.pl/mazda/). Images were reviewed through hospital PACS for all slices and the slice with maximum lesion diameter was exported as .bmp files. The ROI of tumor was drawn by two readers (WHF and XZL) with consensus, which takes about 15 min for one case. During this process, firstly, we choosed one of the four images with the lesion boundary most clearly displayed and drew the ROI manually on this image. Secondly, we copied and pasted the ROI on the other three images and adjusted accordingly. The two-dimensional (2D) region of interest (ROI) was manually placed within the boundary of tumor on each selected image (Fig. 2). In the difficult cases, in which tumors were obscure on CT, MRI was also referenced if it was available. Most patients suspicious of pancreatic tumor underwent both contrast enhanced CT and MRI before surgery in our hospital. There were 124/155 patients, who had preoperative MRI in this study. In addition, the maximum tumor diameter was also recorded for each case.

MaZda software allows computation of almost 300 texture features (Additional file 1) based on image histogram, co-occurrence matrix (COM), run-length matrix (RLM), image gradient, auto-regressive model (ARM) and Haar wavelet (WAV). All of these texture features were extracted from each ROI. To diminish the influence of contrast and brightness variation, we normalized the image grey level intensity in the range of μ-3δ, μ + 3δ (μ, mean grey level value; δ, mean standard deviation). A combination of feature selection algorithms (FPM) including Fisher’s coefficient (Fisher), classification error probability combined with the average correlation coefficients (POE + ACC), and mutual information (MI) was used to determine 30 texture features with the highest discriminative power for differentiation between lymph node metastases positive and negative groups.

### Statistics

The gender of the patients was compared between the two groups using Chi-square test. For the quantitative data with a normal distribution, which presented as mean ± standard deviation, independent samples t-test was used, and for the quantitative data with a skewed distribution, which presented as median (quartile 1, quartile 3), Mann-Whitney U test was used, to evaluate the difference between the two groups. Receiver operating characteristic (ROC) curves were generated for the features that presented significant differences, and the area under the ROC curve (AUC) was calculated and compared between different texture features. The 95% confidence intervals (CI) for AUC and *p* values for comparison of related ROC curves were obtained by the method described by DeLong and coworkers [19]. The relationship between the features (with AUC > 0.5) and LNR were also evaluated with non-parametric Spearman’s correlation coefficients. Values *p* < 0.05 were considered statistically significant. Statistical analysis was performed using SPSS software (SPSS version 23.0; SPSS Inc., Chicago, IL, USA) and MedCalc for Windows, version 11.4 (MedCalc Software, Ostend, Belgium).

## Results

The mean age was 63.3 ± 9.5 years for lymph node positive group (range, 37–83 years, 46 men/26 women) and 64.2 ± 9.8 years for lymph node negative group (range, 40–83 years, 59 men/24 women). The median tumor size of lymph node positive group and lymph node negative group were 3.0 cm (range, 1.0–8.5 cm) and 3.0 cm (range, 1.0–9.5 cm) respectively. There were no statistically significant differences between the two groups for the tumor size, age and gender of the patients (all *p* values > 0.05) (Table 1).

**Table 1:** Comparison of demographic features and tumor size between lymph node metastases positive versus negative pancreatic ductal adenocarcinoma (155 cases)

	Lymph node metastases positive(*n* = 72)	Lymph node metastases negative(*n* = 83)	Comparison between two groups	*P* value
Gender(male/female)	46/26	59/24	Chi-square = 0.614	0.433
Age(mean ± standard deviation), years	63.3 ± 9.5	64.2 ± 9.8	t = 0.613	0.541
Tumor size(Median[quartile 1, quartile 3]), cm	3.0(2.5, 4.0)	3.0(2.5, 4.0)	U = 2803	0.503

Abbreviation: *n* number of cases, *t* independent samples t test, *U* Mann-Whitney test

Categories and numbers of the features selected by MaZda software with FPM method for each image subset were shown in supplementary material (Additional file 2: Table S1). Among them there were 13 features had significant difference between the two groups. The AUCs obtained from the ROC curves were calculated for all 13 significantly different texture features, and 10/13 features had AUC bigger than 0.5 (*p* < 0.05) (Table 2). Among them (10 features with AUC > 0.5) there were six co-occurrence matrix-based features of 1.25 mm-PVP-60 keV image and one co-occurrence matrix-based feature of 5 mm-PVP-65 keV image, two run-length matrix-based features of 5 mm-AP-65 keV image, and one wavelet-based feature of 1.25 mm-AP-60 keV image (Table 2).

**Table 2:** Texture features with significant difference obtained from virtual monochromatic CT images between lymph node metastases positive and negative groups of pancreatic ductal adenocarcinoma (155 cases)

Image subset	Texture feature	N+(n = 72)	N-(n = 83)	U/t	*P* value	AUC(95% CI)	*P* value	Cutoff value	+LR
1.25 mm-AP-60 keV	WavEnLH_s-2^b^	244 (208, 313)	294 (242, 352)	2209	0.005	0.630(0.549–0.706)	0.005	270	2.08
1.25 mm-PVP-60 keV	S(5,-5)SumAverg^a^	62.5 ± 2.0	63.4 ± 2.1	2.657	0.009	0.619(0.538–0.696)	0.009	63.3	1.45
	S(4,-4)SumAverg^a^	62.5 ± 1.8	63.2 ± 1.9	2.303	0.023	0.600(0.518–0.677)	0.029	63.7	1.31
	S(4,4)SumAverg^a^	62.4 ± 1.8	63.0 ± 1.8	2.019	0.045	0.589(0.500–0.679)	0.052	NA	NA
	S(4,0)SumAverg^a^	62.7 ± 1.4	63.2 ± 1.6	1.995	0.048	0.594(0.512–0.672)	0.040	63.4	1.41
	S(3,3)SumAverg^a^	62.6 ± 1.6	63.2 ± 1.6	2.283	0.024	0.607(0.525–0.684)	0.019	62.6	1.67
	S(2,2)SumAverg^a^	62.9 ± 1.3	63.4 ± 1.3	2.216	0.028	0.615(0.534–0.692)	0.012	63.1	1.64
	S(1,1)SumAverg^a^	63.5 ± 0.8	63.7 ± 0.9	2.021	0.045	0.608(0.526–0.685)	0.019	63.6	1.60
5 mm-AP-65 keV	135dr_LngREmph^a^	1.24 ± 0.06	1.22 ± 0.06	−2.133	0.035	0.608(0.526–0.685)	0.019	1.23	1.57
	S(4,-4)SumOfSqs^a^	100 ± 7	97 ± 9	−2.218	0.028	0.573(0.484–0.663)	0.110	NA	NA
	135dr_Fraction^a^	0.929 ± 0.016	0.934 ± 0.015	2.088	0.038	0.612(0.530–0.689)	0.015	0.934	1.58
5 mm-PVP-65 keV	S(5,-5)SumAverg^b^	62.2(61.1, 63.4)	62.8(61.1, 64.6)	2429	0.045	0.594(0.512–0.672)	0.041	63.0	1.36
	S(4,4)SumAverg^a^	62.0 ± 1.8	62.6 ± 2.1	2.047	0.042	0.571(0.481–0.661)	0.121	NA	NA

^a^Data are mean ± standard deviation, independent samples t-test was used

^b^Data are median (quartile 1, quartile 3), Mann-Whitney test was used

Abbreviation: *AP* late arterial phase, *AUC* area under receiver operator characteristic curve, *CI* confidence interval, *keV* kilo electron voltage, *+LR* positive likelihood ratio, *n* number of cases, *N+* lymph node metastasis positive, *N-* lymph node metastasis negative, *NA* not applicable, *PVP* portal venous phase, *SD* standard deviation, *t* independent samples t-test, *U* Mann-Whitney test; 1.25 mm/5 mm, slice thickness of the CT images analysed

**Fig. 3:**
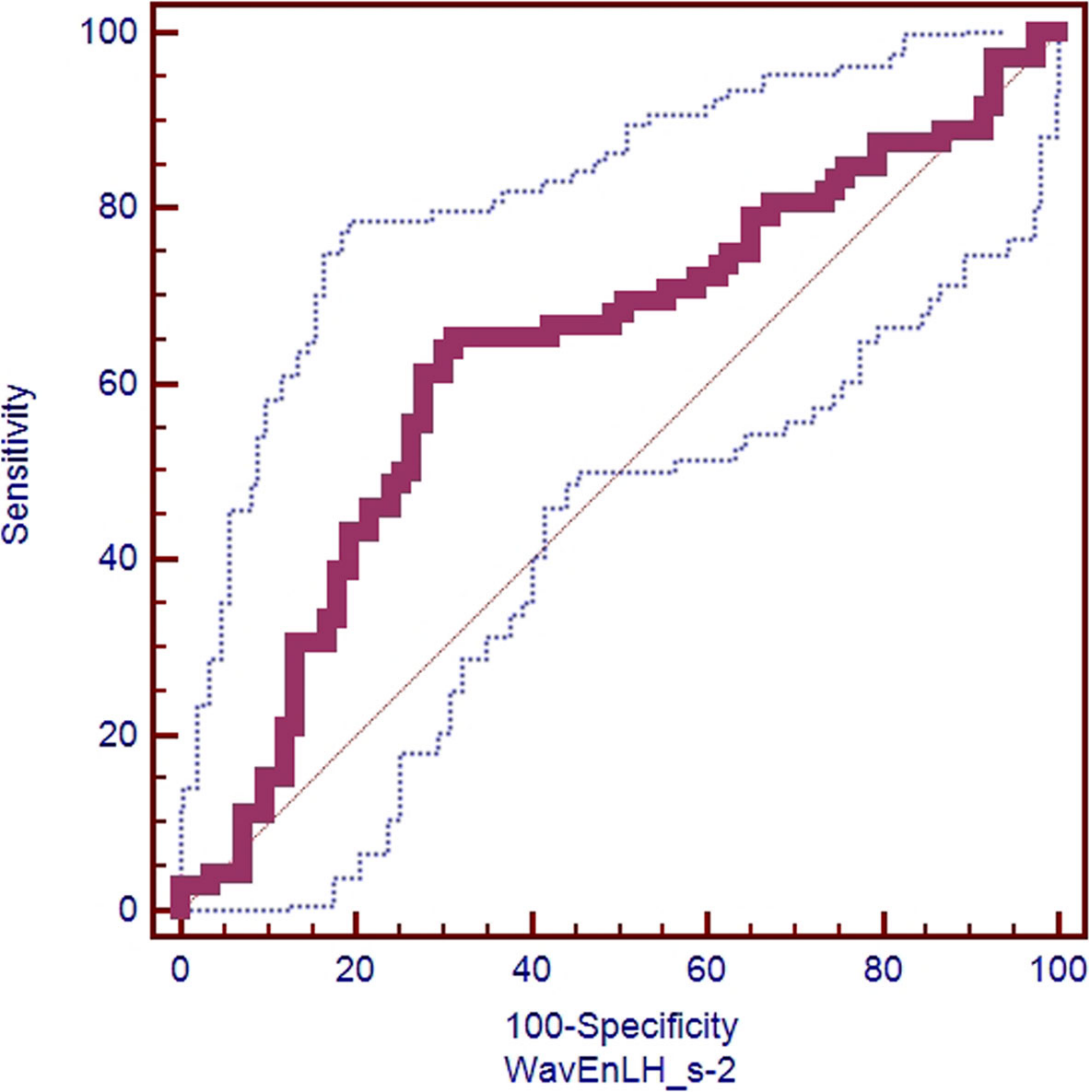
Receiver operating characteristic (ROC) curve of wavelet transform based feature WavEnLH_s-2 (area under ROC curve = 0.630, 95% confidence interval 0.549 to 0.706) from the virtual monochromatic CT image at 60 k electron voltage (keV) on late arterial phase with 1.25 mm slice thickness for differentiating lymph node metastases positive and negative groups of pancreatic ductal adenocarcinoma (155 cases)

Wavelet-based feature WavEnLH_s-2 (wavelet energy with rows and columns are filtered with low pass and high pass frequency bands with scale factors 2) of 1.25 mm-PVP-60 keV image had the biggest AUC (0.630, 95% CI 0.549 to 0.706), but there were no significant differences between the 10 features (all *p* values > 0.05). Ideal cutoff value for predicting LN metastases was 270 for WavEnLH_s-2 (positive likelihood ratio 2.08) according to the ROC analysis (Fig. 3). The lymph node positive group had lower values of WavEnLH_s-2, 135dr_Fraction (fraction of image in runs in 135 direction) from run length matrix based features and sum average from coocurrence matrix based features, and higher value of 135dr_LngREmph (long run emphasis in 135 direction) from run length matrix based features than lymph node negative group.

**Fig. 4:**
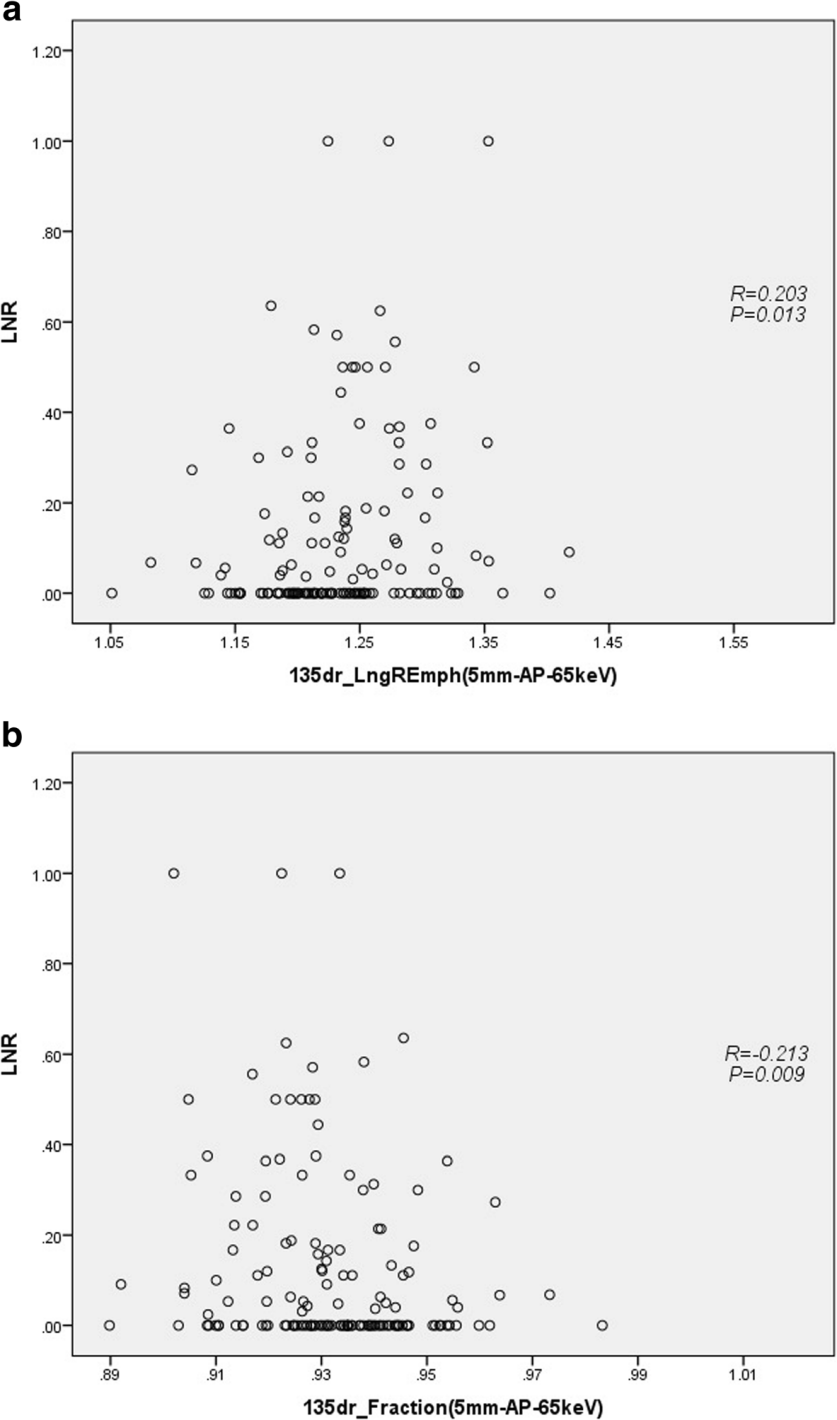
Correlations of ratio of metastatic to examined lymph nodes (LNR) with run-length matrix based features 135dr_LngREmph (**a**) and 135dr_Fraction (**b**) from virtual monochromatic CT images at 65 k electron voltage (keV) on late arterial phase (AP) with 5 mm slice thickness in the pancreatic ductal adenocarcinoma patients (155 cases), analyzed using the non-parametric Spearman’s correlation coefficients method

Among the 10 features, 135dr_LngREmph (*R* = 0.203, *p* = 0.013) and 135dr_Fraction (*R* = -0.213, *p* = 0.009) on 5 mm-AP-65 keV image were associated with the ratio of metastatic to examined lymph nodes (LNR) (Fig. 4). The median LNR was 0.18 (0.09, 0.36) for the lymph node positive patients in this study.

## Discussion

Lymph node metastases is considered a grave prognostic sign in PDAC. Tumor size and lymph node involvement are significantly correlated with OS [2, 20, 21]. Previous study showed that tumor size was found to be an important predictor of lymph node status and predicted odds of positive nodes tend to increase with increasing tumor size [22]. In this series, the median size of tumors was 3.0 cm and there was no significant difference for the tumor size between the lymph node metastases positive and negative groups of PDAC (Table 1). Considering the patients in this study were all locally resectable PDAC, which may result in selection bias for the tumor size.

Texture analysis refers to an objective and quantitative set of metrics calculated for quantifying the textural patterns of images. Previous studies have showed radiomics features were associated with nodal involvement in oncologic imaging [7, 12]. Herein, we studied primary tumor texture features of PDAC at preoperative CT by automatically extracting several quantitative parameters, which were compared according to the nodal involvement status. We concluded that texture analysis was an applicable method with significant differences of texture features between lymph node metastases positive and negative PDAC.

We found that SumAverage (measure of overall image brightness) was the most common co-occurrence matrix-based features after feature selection on 1.25 mm-PVP-60 keV and 5 mm-PVP-65 keV images. Our results suggested that the overall density of CT image at portal venous phase, regardless of the slice thickness, was an important characteristic for predicting of lymph nodes metastases in PDAC. The co-occurrence matrix-based features are second-order histogram features. The co-occurrence matrix can extract statistical information from an image regarding the distribution of pairs of pixels. Theoretically SumAverage tends to identify dispersed patterns of density. Ahmed et al. [23] showed that texture parameter SumAverage from post contrast T1 weighted images of MRI was different in lymph node negative and positive data for breast cancer patients. In our study, the values of SumAverage from portal venous phase images were lower in lymph node positive group than those of negative group. It may suggest that PDAC with overall lower density in virtual monochromatic (VMC) CT images on portal venous phase is more likely to have lymph node metastases. Our results are in keeping with those of previous publications showing the role of texture analysis at CT imaging in the prediction of lymph node involvement. Cassinotto C et al. [13] found that mean attenuation value of the whole tumor (WHOLE-AV) at spatial scaling factor 2 showed significant association with lymph node invasion with OR of 0.886 (CI 0.823–0.955) and an AUC of 0.74 (CI 0.58–0.85).

In addition, our results also showed that wavelet-based feature of WavEnLH_s-2 (wavelet energy with rows and columns are filtered with low pass and high pass frequency bands with scale factors 2) seemed to be the most discriminative features of 1.25 mm-AP-60 keV image. When the cutoff value is <=270, the positive likelihood ratio is 2.08. Wavelet energy calculates the energy from the wavelet-subband coefficients, which is a local energy feature computed using discrete wavelet transform (DWT). DWT analyses the image by decomposing it into a coarse approximation via low-pass filtering and into detail information via high-pass filtering. As an example, LH means rows and columns are filtered with low pass and high pass filter, respectively. Wavelet transform can detect possible structures that maybe present in an image, especially the details of fine structures that cannot be detected with the naked eye. A wavelet-based feature that can amplify the subtle intensity variation between regions and at the same time represent the intensity inhomogeneity within a region. It means that CT image with higher value of wavelet energy has inhomgenous density. In this study, CT image with thin slice at late arterial phase (1.25 mm-AP-60 keV) had lower value of WavEnLH_s-2 in the lymph node positive group than in the lymph node negative group, which may suggest that PDAC with more homogenous density in VMC CT image at late arterial phase is more likely to have lymph node metastases.

The gray-level run-length matrix (RLM) method is a method of extracting higher order statistical texture features, which is designed to characterize the regions with the same gray-level in the tumor. As the second-order texture features, RLM-based features describe the gray-level relationship of the nearby pixel pairs, thereby reflecting the spatial distribution of pixels in a local way. Run-length matrix -based features indicate the coarseness of a texture in a predetermined direction. The nonuniformity parameters and short-run emphasis are measures of heterogeneity; long-run emphasis (LngREmph) describes the uniformity, and fraction measures local variation in the image. Higher value of long-run emphasis means more unform of image gray-level, while higher value of fraction means bigger variation of image gray-level. In this study, CT image with thick slice at late arterial phase (5 mm-AP-65 keV) had higher value of 135dr_LngREmph (long run emphasis) and lower value of 135dr_Fraction (fraction of image in runs) in the lymph node positive group than in the lymph node negative group, which may suggest that PDAC with uniform density on late arterial phase VMC CT image (5 mm-AP-65 keV) is more likely to have lymph node metastases.

According to the previous studies [20, 24, 25], the ratio of metastatic to examined lymph nodes (LNR) was an optimal prognostic indicator for short- and long-term survival after surgery for PDAC. LNR was found to have a highly significant, non-linear effect on survival [22]. In this study, there were 10 features that had significant differences between the two groups. Among these features, only the run-length matrix-based features 135dr_LngREmph and 135dr_Fraction on 5 mm-AP-65 keV image were associated with LNR of PDAC. Run-length matrix-based feature (5 mm-AP-65 keV image) 135dr_LngREmph had positive correlation (*r* = 0.203, *P* = 0.013) and 135dr_Fraction had negative correlation with LNR (*r* = − 0.213, *P* = 0.009). The higher value of 135dr_LngREmph and lower value of 135dr_Fraction suggest a higher risk of nodal involvement, which further confirmed the aforementioned results of comparison between two groups. These results suggested that PDAC with uniform density on late arterial phase CT image is more likely to have lymph node metastases.

The present study has several limitations. First, this is a single center study with small sample size. Given that our study was a single center study with standardized and uniform CT imaging parameters, our results might not apply to other institutions, in particular those that use different CT imaging techniques. Second, we performed texture analysis only on 2D images by selecting the section showing the maximum diameter of the tumor. Although this is a standard approach used in many previous studies, it may be less representative of the entire tumor volume than 3D analysis. 2D approach is sill widely used because of its operability and practicality [12, 26]. Third, inter-observer variability was not available in this study. The inter-observer variability for research with any manual operation involvement is an open problem, and we think that it could be conducted in further study. Finally, the statistical associations between texture parameters and the lymph nodes status we found in our work were obtained only from a selected study population. In the future, studies focusing on texture features should be conducted in validation populations and in other centers with the use of different CT imaging techniques to confirm our findings. It would also be of interest to investigate additional software allowing 3D texture analysis. Despite these drawbacks, we believe our study may be valuable.

## Conclusions

The results of our study showed that texture features, especially second and high order texture features, obtained from preoperative CT image of primary tumor had differences between lymph node metastases positive and negative groups and the potential to predict the nodal involvement of PDAC. Tumor with homogenous/uniform density on late arterial phase and overall lower density on portal venous phase in preoperative CT images is more likely to have lymph node metastases.

## Supplementary information

**Additional file 1.** Texture parameters computed by MaZda

**Additional file 2:Table S1.** Categories and numbers of the features selected by MaZda software with FPM method for each image subset

## Data Availability

The datasets used and/or analysed during the current study are available from the corresponding author on reasonable request.

## Abbreviations

2D: Two-dimensional
3D: Three-dimensional
AP: Late arterial phase
AUC: Area under receiver operating characteristic curve
CI: Confidence interval
COM: Co-occurrence matrix
CT: Computed tomography
DWT: Discrete wavelet transform
FPM: A combination of feature selection algorithms including Fisher’s coefficient (Fisher), classification error probability combined with the average correlation coefficients (POE+ACC), and mutual information (MI)
LN: Lymph node
LNR: Ratio of metastatic to examined lymph nodes
N-: Lymph node metastases negative
N+: Lymph node metastases positive
OS: Overall survival
PVP: Portal venous phase
RLM: Run-length matrix
ROC: Receiver operating characteristic
ROI: Region of interest
VMC: Virtual monochromatic
WAV: Wavelet
