# Supplementary material for: Resectable pancreatic ductal adenocarcinoma: association between preoperative CT texture features and metastatic nodal involvement
Source: Cancer Imaging. 2020 Feb 10;20:17. doi: 10.1186/s40644-020-0296-3 (PMC7011565; doi:10.1186/s40644-020-0296-3)
Supplement: Supplementary file 1 — Additional file 1. Texture parameters computed by MaZda [file 40644_2020_296_MOESM1_ESM.docx]

Supplementary material

Texture parameters computed by MaZda

1. Histogram

Mean (histogram’s mean)

Variance (histogram’s variance)

Skewness (histogram’s skewness)

Kurtosis (histogram’s kurtosis)

Perc.01% (1% percentile)

Perc.10% (10% percentile)

Perc.50% (50% percentile)

Perc.90% (90% percentile)

Perc.99% (99% percentile)

Total number of histogram based features: 9

2. Gradient

GrMean (absolute gradient mean)

GrVariance (absolute gradient variance)

GrSkewness (absolute gradient skewness)

GrKurtosis (absolute gradient kurtosis)

GrNonZeros (percentage of pixels with nonzero gradient)

Total number of absolute gradient based features: 5

3. Run Length Matrix

RLNonUni (run length nonuniformity)

GLevNonU (grey level nonuniformity)

LngREmph (long run emphasis)

ShrtREmp (short run emphasis)

Fraction (fraction of image in runs)

Features are computed for 4 (2D images) various directions.

Total number of run length matrix based features: 20 (2D)

4. Coocurrence Matrix

AngScMom (angular second moment)

Contrast (contrast)

Correlat (correlation)

SumOfSqs (sum of squares)

InvDfMom (inverse difference moment)

SumAverg (sum average)

SumVarnc (sum ariance)

SumEntrp (sum entropy)

Entropy (entropy)

DifVarnc (difference variance

DifEntrp (difference entropy)

Features are computed for 5 between-pixels distances (1, 2, 3, 4, 5) and for 4 (2D images) various directions.

Total number of co-occurrence matrix based features: 220 (2D)

5. Autoregressive Model

Teta1 (parametr q1)

Teta2 (parametr q2)

Teta3 (parametr q3)

Teta4 (parametr q4)

Sigma (parametr s)

Total number of autoregressive model based features: 5

6. Haar Wavelet

WavEn (wavelet energy)

Feature is computed at 5 scales within four frequency bands LL, LH, HL and HH.

Total number of Haar wavelet based features: 20
