# Supplementary material for: Resectable pancreatic ductal adenocarcinoma: association between preoperative CT texture features and metastatic nodal involvement
Source: Cancer Imaging. 2020 Feb 10;20:17. doi: 10.1186/s40644-020-0296-3 (PMC7011565; doi:10.1186/s40644-020-0296-3)
Supplement: Supplementary file 2 — Additional file 2:Table S1. Categories and numbers of the features selected by MaZda software with FPM method for each image subset [file 40644_2020_296_MOESM2_ESM.docx]

Table 1 Categories and numbers of the features selected by MaZda software with FPM method for each image subset

|  | Image histogram | Image gradient | Run-length matrix | Co-occurrence matrix | Auto-regressive model | Haar wavelet |
| --- | --- | --- | --- | --- | --- | --- |
| 1.25mm-AP-60keV | 2 | 1 | 1 | 20 | 3 | 3 |
| 1.25mm-PVP-60keV | 2 | 0 | 0 | 23 | 2 | 3 |
| 5mm-AP-65keV | 1 | 2 | 5 | 20 | 2 | 0 |
| 5mm-PVP-65keV | 1 | 1 | 0 | 25 | 1 | 2 |

Data are the numbers of the selected texture features.

Abbreviation: AP, late arterial phase; FPM, A combination of feature selection algorithms including Fisher’s coefficient (Fisher), classification error probability combined with the average correlation coefficients (POE+ACC), and mutual information (MI); keV, kilo electron voltage; PVP, portal venous phase; 1.25mm/5mm, slice thickness of the virtual monochromatic CT images analysed.
